# Biplanar Low-Dose Radiograph Is Suitable for Cephalometric Analysis in Patients Requiring 3D Evaluation of the Whole Skeleton

**DOI:** 10.3390/jcm10235477

**Published:** 2021-11-23

**Authors:** Adeline Kerbrat, Isabelle Rivals, Pauline Dupuy, Gauthier Dot, Britt-Isabelle Berg, Valérie Attali, Thomas Schouman

**Affiliations:** 1 Service de Chirurgie Maxillo-Faciale, Hôpital Pitié-Salpêtrière, AP-HP Sorbonne Université, 75013 Paris, France; dupuypaulinebdx@gmail.com (P.D.); thomas.schouman@aphp.fr (T.S.); 2 Arts et Metiers ParisTech, LBM/Institut de Biomécanique Humaine Georges Charpak, 75013 Paris, France; gauthier.dot@ensam.eu (G.D.); valerie.attali@aphp.fr (V.A.); 3 Sorbonne Université, Inserm, UMRS1158 Neurophysiologie Respiratoire Expérimentale et Clinique, 75005 Paris, France; isabelle.rivals@espci.fr; 4 Equipe de Statistique Appliquée, ESPCI Paris, PSL Research University, 75231 Paris, France; 5 Department of Cranio-Maxillofacial Surgery, University Hospital Basel, CH-4031 Basel, Switzerland; isabelle.berg@usb.ch; 6 Service des Pathologies du Sommeil, Département R3S, Hôpital Pitié-Salpêtrière, AP-HP. Sorbonne Université, 75013 Paris, France

**Keywords:** biplanar X-ray, cephalometric analysis, lateral cephalograms, reliability

## Abstract

Background: The biplanar 2D/3D X-ray technology (BPXR) is a 2D/3D imaging system allowing simultaneous stereo-corresponding posteroanterior (PA) and lateral 2D views of the whole body. The aim of our study was to assess the feasibility of cephalometric analysis based on the BPXR lateral skull view to accurately characterize facial morphology. Method: A total of 17 landmarks and 11 angles were placed and/or calculated on lateral BPXR and lateral cephalograms of 13 patients by three investigators. Five methods of angle identification were performed: the direct construction of straight lines on lateral cephalograms (LC-A) and on BPXR (BPXR-A), as well as the calculation of angles based on landmark identification on lateral cephalograms (LA-L) and on BPXR with the PA image (BPXR-LPA) or without (BPXR-L). Intra- and interoperator reliability of landmark identification and angle measurement of each method were calculated. To determine the most reliable method among the BPXR-based methods, their concordance with the reference method, LC-A, was evaluated. Results: Both imaging techniques had excellent intra- and interoperator reliability for landmark identification. On lateral BPXR, BPXR-A presented the best concordance with the reference method and a good intra- and interoperator reliability. Conclusion: BPXR provides a lateral view of the skull suitable for cephalometric analysis with good reliability.

## 1. Introduction

Cephalometric analysis based on lateral (and frontal) cephalograms is commonly used by orthodontists and maxillofacial surgeons to complete clinical diagnosis in patients with maxillomandibular deformities (MMDs) and to characterize upper airways in patients with obstructive sleep apnea syndrome (OSAS) before maxillo-mandibular advancement osteotomy or treatment with mandibular advancement splint. Numerous analyses have been described [1,2] to characterize the jaw’s deviation from its theoretical position. Lateral cephalograms are routinely used in the assessment of MMDs to establish a treatment plan and during follow-up. Traditional cephalometric analysis is based on landmarks and lines drawn on acetate overlays or on digitalized X-ray images, and on further calculations of specific linear and angular indices between these landmarks [3].

The biplanar 2D/3D X-ray technology (BPXR) (EOS imaging^®^, Paris, France) based on a low-dose X-ray system allows simultaneous stereo-corresponding posteroanterior (PA) and lateral 2D images of the whole body, including the head, to be taken in a calibrated environment [4,5,6]. BPXR uses an ultrasensitive multiwire proportional chamber detector to detect X-rays, thus limiting the dose of X-rays absorbed by the patient. The EOS 2D/3D imaging system allows the 3D reconstruction of the whole skeleton based on two-dimensional X-rays acquired in an upright position. This system provides reliable 2D images of the spine and is validated for clinical practice in orthopedics [7,8]. For example, BPXR is used to follow up patients with idiopathic scoliosis treated with the in-brace method [9]. In patients with scoliosis, numerous X-ray images are taken to follow the patient’s growth or the impact of the treatment on the spine alignment [10,11,12]. With low irradiation, BPRX provides a useful tool for the radiological follow-up of patients with spinal deformities, especially children.

Body posture and facial skeleton typology seem to be connected [13]. Scoliosis, for instance, is frequently associated with jaw asymmetry [14]. In this pathology, the body posture disorder most likely impacts the development of the facial skeleton through the muscular dysfunction of the trunk. Conversely, cervical spine hyperextension and subsequent postural disorder are observed in patients with MMDs causing mouth breathing, as in patients with OSAS, despite the absence of postural disorder history for most of these patients [15]. In both cases, the hyperextension of the neck results from an adaptation of the posture to fight increased upper away resistance and/or instability [16,17]. BPXR represents a promising tool to investigate the interaction between posture and facial typology. Berg et al. demonstrated that BPXR imaging of the facial skeleton was sufficient for landmark assessment [18]. However, the lateral view of BPXR has never been used for cephalometric assessment. If cephalometric analysis could be obtained using BPXR lateral skull view, the facial morphology of patients using BPXR for scoliosis or another spinal condition could be evaluated without additional X-ray exposure.

The aim of our study was to assess the feasibility of cephalometric analysis based on BPXR lateral skull X-ray images to accurately characterize facial morphology.

## 2. Materials and Methods

### 2.1. Subjects

The imaging data of 22 patients who underwent both lateral cephalograms and BPXR as part of a clinical study investigating their posture were collected in the department of maxillofacial surgery of the Pitié-Salpêtrière Hospital. The study included 14 patients presenting with OSAS (ISRCTN70932171) and 8 patients with an MMD (NCT03532828). This study received approval from the relevant ethics committees (Comité de Protection des Personnes (CPP) Sud-Méditerranée 2018-A00362-53 and CPP Ile de France VI 2006-A00386-45). All patients provided written informed consent. 

Eight patients were excluded because their lateral cephalograms were not digitalized and could not be imported in digital imaging and communication in medicine (DICOM) format. One patient was excluded because his head was incomplete on the EOS lateral view. We analyzed the data of the remaining 13 patients.

### 2.2. Image Acquisition

Two biplanar perpendicular radiographs were obtained simultaneously using the EOS imaging system in a standardized position, with the hands resting on the cheeks (the so-called Scoliosis Research Society modified free-standing position) and the feet positioned as described by Chaıbi et al. [19]. BPXR provides two simultaneous orthogonal views: a posteroanterior (PA) view and a lateral view. The lateral cephalometric radiographs were obtained using a digital cephalometric device (Planmeca Promax, Planmeca^®^, Helsinki, Finland). The cephalograms and the BPXR were achieved on two different days.

### 2.3. Comparison of BPXR and Lateral Cephalograms

**Figure 1:**
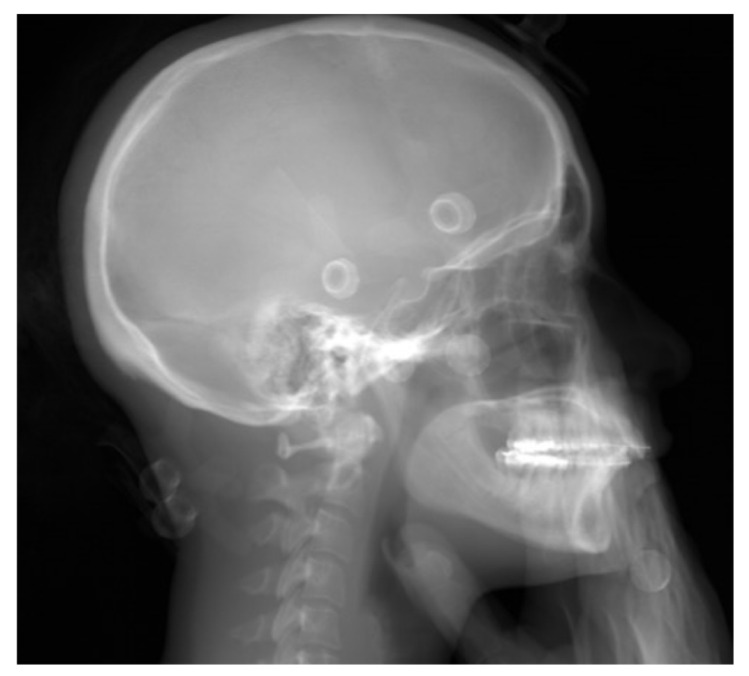
Skull lateral BPXR view.

**Figure 2:**
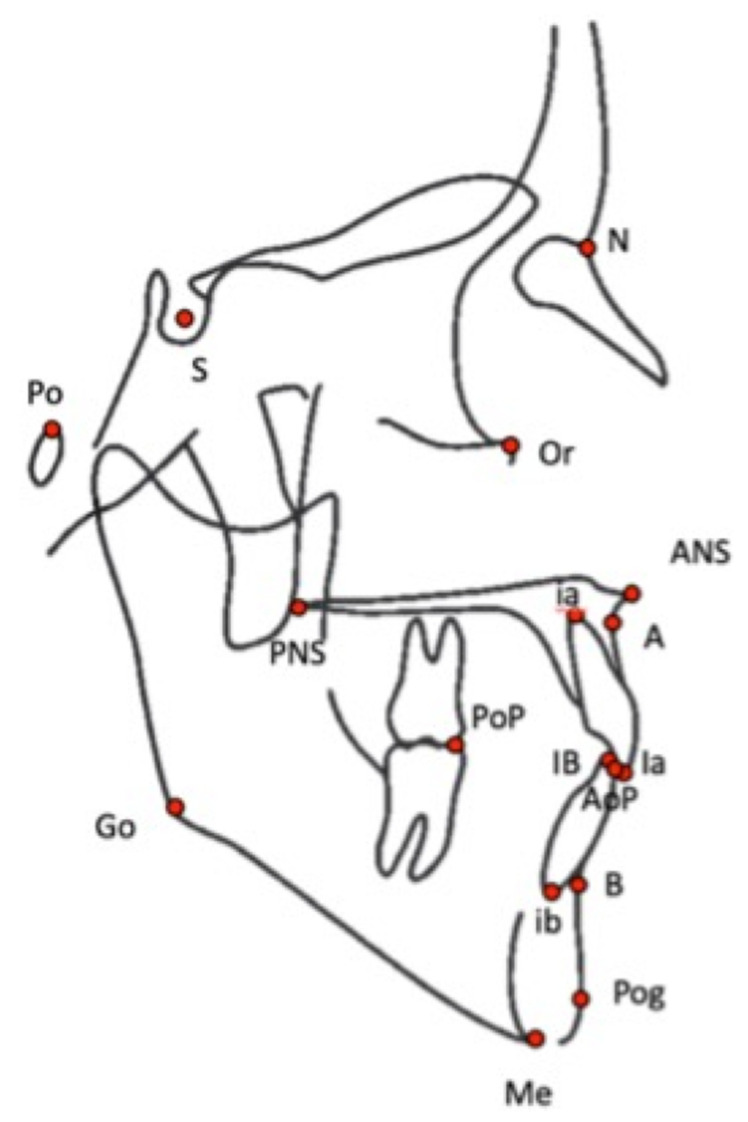
Landmarks used to perform the cephalometric analysis. S: center of the sella turcica, N: N = nasion, ANS: anterior nasal spine, PNS: posterior nasal spine, A: most posterior point of the anterior contour of the upper alveolar process, B: most posterior point of the anterior contour of the lower alveolar process, Me: most inferior point of the chin, Po: porion, Or: orbital, Pg: most anterior point on the mandibular symphysis, Go: gonion (a bisecting point between the mandibular plane and the posterior edge of the ramus), AI: mid-point of the incisal edge of the most prominent upper central incisor, BI: mid-point of the incisal edge of the most prominent lower central incisor, ai: apex of the root of the upper central incisor, bi: apex of the root of the lower central incisor, AoP: anterior occlusal point, PoP: posterior occlusal point.

To compare the two types of images, we used common cephalometric angles (see Table 1). DICOM images of the BPXR of the skull (Figure 1) and lateral cephalograms were imported in IdefX (v5.1.0, Paris, France). IdefX is a software developed to process BPXR images to generate 3D models from the two orthogonal biplanar X-rays. The landmarks used for the analysis are shown in Figure 2. The coordinates of the landmarks are obtained in two different geometrical frames. Indeed, the origin is different in the two radiographs. With BPXR, head-to-feet images were acquired while lateral cephalograms contained the skull and the cervical spine, each of them corresponding to a specific coordinate system. Therefore, we could not compare the coordinates of the landmarks obtained by the 2 methods. Angles obtained from landmark positioning were recorded. For BPXR, the angles were obtained in three different ways as follows:

(1) Angles between pairs of lines constructed from the landmark coordinates on the BPXR lateral view (BPXR-L);
(2) Angles between pairs of lines constructed from the landmark coordinates on the BPXR lateral view, as marked with the help of the corresponding PA view (a line indicates the corresponding height of the landmark on the PA view when the landmark is positioned on the lateral view in Idefix; BPXR-LPA);
(3) Angles between pairs of lines drawn directly on the BPXR lateral view, without previous landmark identification (BPXR-A).

**Table 1:** Angles used in our cephalometric analysis.

Angle	Definition
SNA	Angle between S, N, and A points
SNB	Angle between S, N, and points
ANB	Angle between A, N, and B points
FMIA	Angle between Frankfort (Or-Po) plane and mandibular incisor axis (Bi-bi)
FMA	Angle between Frankfort plane and mandibular plane (Go-Me)
IMPA	Angle between mandibular incisor axis and mandibular plane
I/SN	Angle between maxillary incisor axis (Ia-ia) and S-N plane
I/i	Angle between maxillary incisor axis and mandibular incisor axis
SN/Pocc	Angle between S-N plane and occlusal plane (AoP-PoP)
Fr/Pocc	Angle between Frankfort plane and occlusal plane
Max/Mand	Angle between maxilla plane (ENA-ENP) and mandibular plane
**Distance**	**Definition**
S-N	Distance between S and N
ANS-PNS	Distance between ANS and PNS
Me-IB	Distance between Me and IB
N-ANS	Distance between N and ANS

On lateral cephalograms, the cephalometric analysis and angle calculation were performed following two different methods: with the construction of straight lines (LC-A) and using landmarks (LC-L). The concordance of the three methods of BPXR with the reference method (LC-A) was then evaluated in order to determine the most reliable BPXR method.

To assess possible differences in the distortion, we also compared the two types of images based on the measurement of two horizontal and two vertical distances between pairs of landmarks, as listed in Table 1.

For each method, in order to estimate intra- and interoperator reliability, the angle measurement was performed by the three operators, repeated three times for operators 1 and 2, and two times for operator 3, with a minimum 2-week interval. A training session was organized 2 weeks before taking the measurements. All measurements were performed using IdefX.

### 2.4. Statistical Analysis

#### 2.4.1. Intraoperator Reliability

For each of the three operators, the intraoperator (i.e., test–retest) reliability was investigated (1) for the landmarks (for the three methods using landmarks, i.e., LC-L, BPXR-L, and BPXR-LPA) and (2) for the angles (for the five methods). This reliability was evaluated using an intraclass correlation coefficient ICC(A,1) according to the notation used by McGraw and Wong [20]. ICC(A,1) is the variance ratio quantifying the absolute agreement between the two or three measurements of an operator for each of the 13 patients (two-way ANOVA model). According to Koo and Li, ICC values less than 0.5, between 0.5 and 0.75, between 0.75 and 0.9, and greater than 0.9 are indicative of poor, moderate, good, and excellent reliability, respectively [21]. Confidence intervals were estimated as in Mc Graw and Wong [20]. To summarize the results, mean ICCs over operators were taken over the Fisher transform (i.e., ln((1 + r)/(1 − r))) of the individual ICCs, and transformed back.

#### 2.4.2. Interoperator Reliability

The interoperator reliability was investigated (1) for the landmarks (for the three methods using landmarks, i.e., LC-L, BPXR-L, and BPXR-LPA) and (2) for the angles (for the five methods). This reliability was also evaluated using ICC(A,1). Here, the variance ratio quantified the absolute agreement between a single measurement of the three operators for each of the 13 patients. For each patient, a single measurement of each operator was randomly chosen from among the two or three they made. The means of ICCs for the angles or methods were taken using the Fisher transform.

#### 2.4.3. Concordance with the Reference Method

For each operator, the concordance with the reference method (LC-A) was investigated for each of the four other angle identification methods. This concordance was also evaluated using ICC(A,1); here, the variance ratio quantified the absolute agreement between an operator’s measurement using the method of interest and that obtained with the reference method, for each of the 13 patients. To synthesize the ICCs of the three operators, means were taken using the Fisher transform.

#### 2.4.4. Linear Distances

The mean, standard deviation (SD) of the difference between the repeated measurements for each method and between the two methods were calculated. The reproducibility was calculated by paired measurement comparisons with a *t*-test. The level of statistical significance was set at *p* < 0.05.

The statistical analysis was performed with MATLAB (version 9.10.0.1684407 (R2021a) update 3).

## 3. Results

### 3.1. Landmarks

We found excellent intraoperator reliability for most of the landmarks. All landmarks had an intrarater ICC > 0.9 regardless of the method. The results are displayed in Figure S1. The interoperator reliability was also excellent. The mean interrater ICC was above 0.9 for all landmarks except “x-Or” (0.88) and “x-PoP” (0.76) for the LC-L method. The results are displayed in Figure S2.

### 3.2. Distances

Mean differences between the LC-method and the BPXR-method were 0.22 mm (+/− 1.41, *p* = 0.6) for N-S, 1.24 mm (+/− 2.33, *p* = 0.20) for N-ANS, 1.75 mm (+/− 2.0, *p* = 0.22) for ANS-PNS and 1.00 mm (+/− 1.6, *p* = 0.18) for Me-Ib. No significant differences were observed on linear distances. The results are displayed in Table S1.

### 3.3. Angles

**Figure 3:**
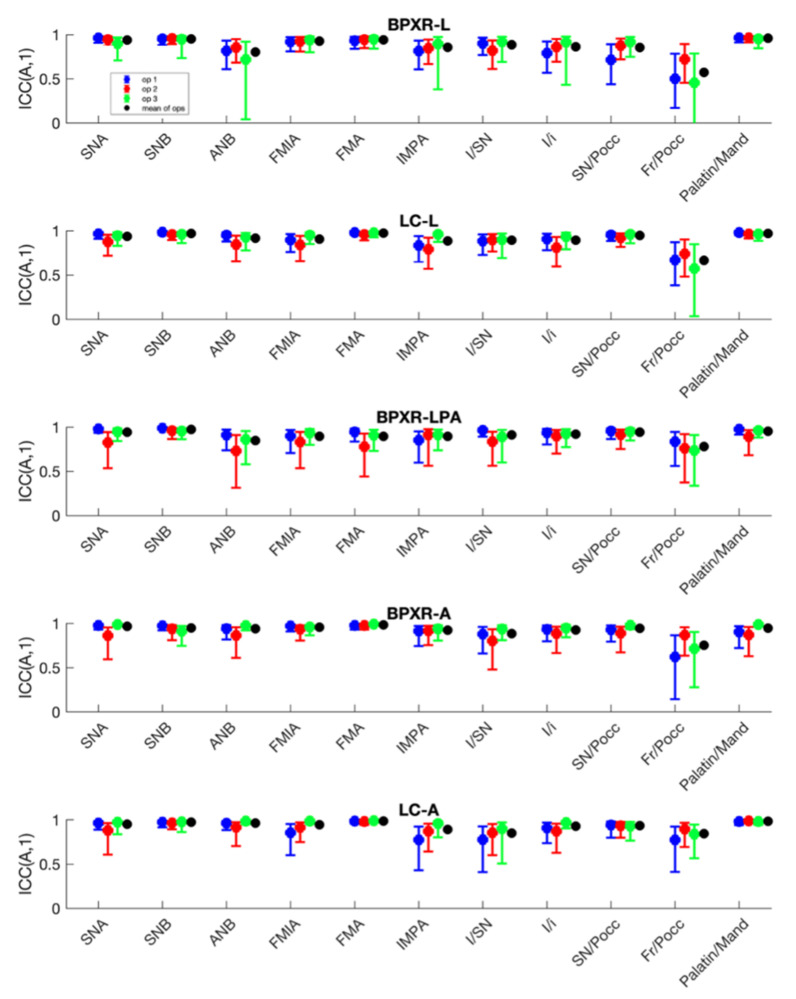
Intraoperator reliability of each angle according to the method.

Intraoperator reliability was better for the LC-A method (mean ICC of 0.95 for the three operators and all angles) than for the LC-L method (ICC = 0.93). The results are displayed in Figure 3. On the BPXR lateral view, the angles measured between straight lines (BPXR-A) presented better results (mean ICC for all angles = 0.94) than those acquired based on landmark identification; the mean ICC was 0.92 for the BPXR-LPA method and 0.90 for the BPXR-L method.

**Figure 4:**
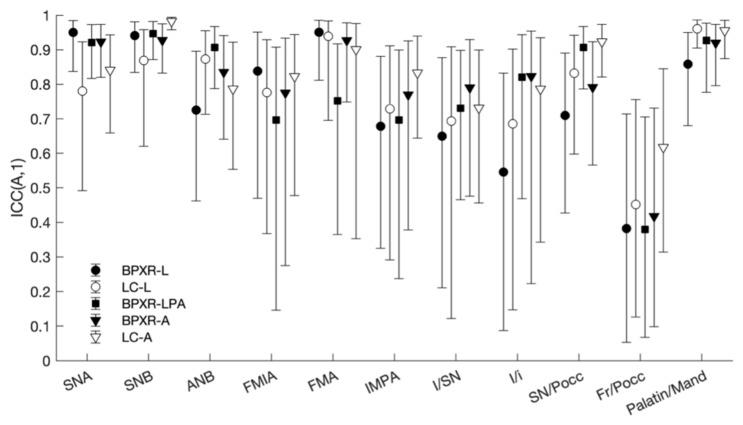
Interoperator reliability of each angle according to the method.

The interoperator reliability was better for the LC-A method (mean ICC of 0.87 for the three operators) than for the LC-L method (mean ICC = 0.83). The results for each angle are displayed in Table 2 and Figure 4. For the lateral cephalogram, with the construction of straight lines to conduct the analysis, all angles showed good or excellent reliability except for the Fr/Pocc and I/SN angles. For the LC-A method, four angles presented poor or moderate reliability. For BPXR, the reliability was best with the construction of straight lines (BPXR-A). Only one angle presented poor or moderate reliability with this method (mean ICC of 0.42 for Fr/Pocc). The reliability was poor or moderate for five angles with the BPXR-LPA method and for four angles with the BPXR-L method.

**Figure 5:**
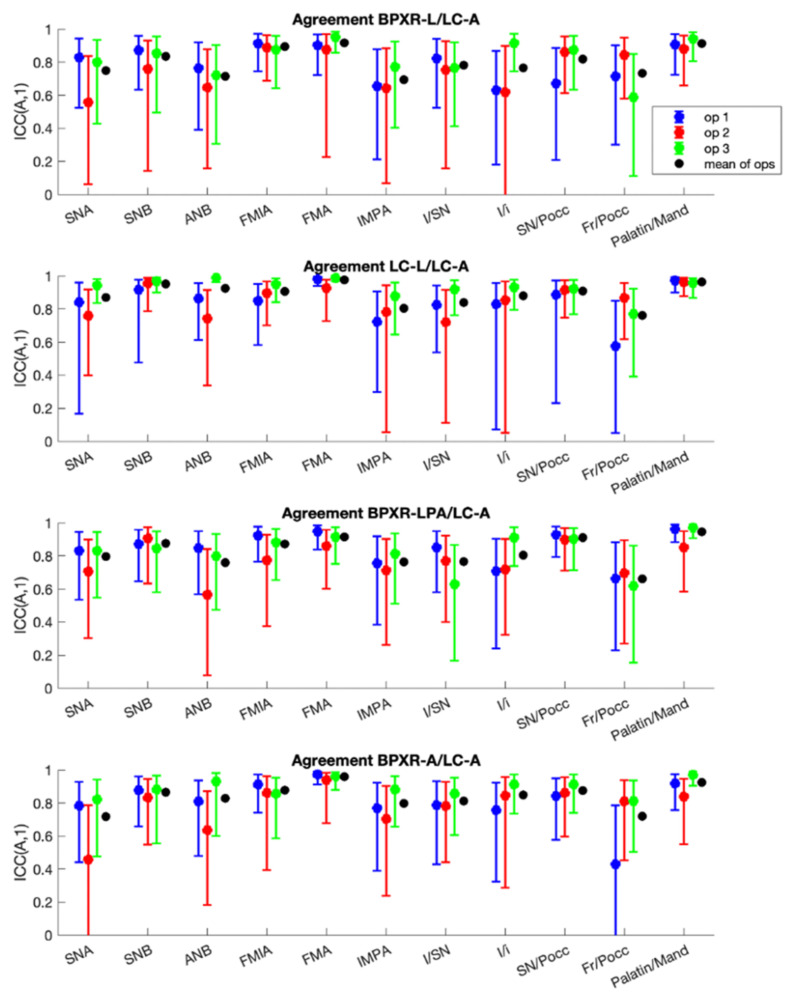
Concordance of each method with the LC-A method (reference method).

The concordance with the reference method was the best with the BPXR-A method, as presented in Figure 5 and Table 3. With the BPXR-A method, the mean ICC of the angles was 0.86, while it was 0.84 for the BPXR-LPA method and 0.82 for the BPXR-L method. The concordance with the reference method was good or excellent for most angles, except Fr/SN (mean ICC = 0.72).

## 4. Discussion

To our knowledge, this study is the first to assess the intra- and interobserver reliability of three methods for cephalometric analysis from the lateral radiography results obtained by BPXR. The best concordance with the reference method (based on a lateral cephalogram) was achieved by measuring angles of interest via the construction of straight lines on BPXR lateral views.

Methods to acquire BPXR and lateral cephalometric radiographs are inherently different. BPXR is mostly used to provide reliable images of the spine and the entire skeleton, while lateral cephalometric radiography is dedicated to cephalometric evaluation. BPXR is a new slot-scanning radiologic device that allows the acquisition of two X-ray images simultaneously. It is composed of two X-ray sources, shaped as fan beams through collimation slits [22]. The core of the BPXR imaging system is a multiwire proportional chamber with two independent X-ray tubes, each producing a 45 cm wide X-ray fan beam and X-ray detection plates [23]. The co-linked X-ray tube/detector pairs run from the top of the head to the feet concurrently in both planes (anterior–posterior and lateral), resulting in two acquisitions in one mechanical motion. Throughout the X-ray tube/detector pair, the X-ray beam is orthogonal to the object being radiographed; in this way, possible parallax deformations of the image are avoided [24]. A lateral cephalometric radiograph is a standardized, reproducible radiograph taken from a distance of 1.5 m with the head at a right angle to the X-ray beam. The X-ray detector is located 15 cm from the head. The cephalostat machine incorporates two posts that are placed in the external auditory meatus, while the patient’s sagittal plane should be parallel to the X-ray film; the teeth are in centric occlusion, and the Frankfort plane is aligned horizontally. As the X-rays emanating from the source have a divergent pattern, the amount of magnification of the object will vary in the radiograph [25]. Manufacturers of scanning digital cephalometric units incorporate proprietary algorithms following image acquisition that correct for image distortion produced by potentially aberrant beam geometries [26]. Magnification can also have an impact on the measurement. On BPXR, magnification is close to zero, as demonstrated by Chiron et al. [27]. On lateral cephalogram, a scale is provided to ensure that the image is at a 1:1 scale. We also compared linear distances to investigate the possible impact of distortion or magnification on our results: no significant differences were observed. The head position during the image acquisition also differs between the two imaging techniques. For a lateral cephalogram, the head is positioned precisely horizontally using a cephalostat (a head-positioning device used in dentomaxillofacial radiology), maintaining the head strictly orthogonal to the X-ray beam to obtain a “true profile”. With this acquisition technique, high reproducibility of the head positioning is expected. However, the reproducibility of the head and cervical postures is poor [24]. During the acquisition with BPXR, the patient stands in a standardized position, with the hands resting on the cheeks without any device to maintain the head. The patient is asked to look into the horizon and to stay still. Consequently, the position of the head is free in order to have the most natural body posture. Even though the patient is placed in alignment with the PA X-ray source and orthogonal to the lateral with the help of footprint stickers on the floor and reference lines on the walls of the cabin, the lateral image of the head may not be a perfect “true profile”. These differences could have impacted our results. However, it seems that the impact on the concordance between the two imaging methods was small, even though acquisition variability assessment was not carried out in this study.

BPXR offers the advantage of allowing 3D reconstructions of the spine, including the cervical spine, with a higher reproducibility of sagittal balance than with 2D imaging [28]. In this setting, BPXR is used in routine practice for treatment decisions during scoliosis follow-up. Patients with scoliosis undergo numerous BPXRs to investigate the evolution of their spinal condition. The incidence of malocclusion in this population is higher than in scoliosis-free people [29]. In addition, postural dysfunction seems to be connected to diseases associated with cephalometric abnormality and mouth breathing such as MMDs [13] or upper airway instability such as OSAS [13,16]. Consequently, the use of BPXR could provide a concurrent evaluation of postural and cephalometric abnormalities, which are often linked [13], while limiting X-ray exposure.

The EOS standardized position, as described by Chaıbi et al., is a standing position with the hands resting on the cheeks [19]. It increases the superimposition of anatomical structures and thus the difficulty in landmark positioning, especially for dental landmarks. It is well known that the superimposition of elements is a source of error in cephalometric analysis and landmark positioning [30]. In our study, the intra- and interoperator reliability of angle measurement were better with the lateral cephalogram. However, BPXR lateral view analysis with the direct drawing of lines was also associated with good to excellent intra- and interoperator reliability. There was a negligible impact on the analysis of the superimposition of the hands resting on the mandible. This result is consistent with those of Berg et al., who found no significant difference in the accuracy of landmark positioning with or without hand superimposition on the facial skeleton [18].

In our study, the inter- and intraoperator reliability of landmark positioning were satisfactory for both imaging technics. In the literature, only one study conducted a similar evaluation on the facial skeleton. Berg et al. evaluated the feasibility of precisely assessing anatomical landmarks of the facial skeleton [18]. The landmarks were precisely detectable, even when patients had their hands in front of the face. However, in our study, the landmarks with both imaging techniques were obtained in two different geometrical frames. The lateral cephalogram view contains only the skull and the cervical spine, whereas BPXR includes the entire skeleton. The y-coordinate is therefore not consistent. For this reason, we compared the angles between the two imaging technics in order to evaluate the feasibility and accuracy of the cephalometric analysis. 

The BPXR images presented another difficulty related to the low dose acquisition—the contrast in the BPXR images was less than in the lateral cephalograms. Some landmarks, especially dental landmarks, were difficult to identify on BPXR images. However, some dental landmarks (especially dental apices) are known to be more difficult to identify than skeletal landmarks. The location of the apices is frequently based on general knowledge of the expected rate of taper perceived from the crown and the visible portion of the root. This difficulty has been highlighted by several authors [31,32]. In our study, the less reliable angles were those involving dental landmarks: the I/SN and Fr/Pocc angles. To assess facial morphology on BPXR, skeletal angles should be preferred.

Our study has some limitations., We focused on group mean assessments in order to evaluate the feasibility of performing a cephalometric analysis on BPXR, but we did not test differences in individual cases. Thus, the outcomes are only applicable in groups of patients (e.g., study groups) and not in individual patients.

## 5. Conclusions

BPXR provides a lateral view of the skull suitable for cephalometric analysis with good reliability compared with cephalometric analyses based on lateral cephalograms. In patients requiring repeated postural evaluation with BPXR imaging, such as scoliotic patients or those with OSAS or MMD and related postural dysfunction, concomitant cephalometric and postural analysis is feasible, thereby avoiding additional X-ray irradiation. 

## Figures and Tables

**Table 2:** Interoperator reliability for angle measurements evaluated with ICCs.

Angle	LC-A ^1^	BPXR-A ^2^	BPXR-LPA ^3^	LC-L ^4^	BPXR-L ^5^	Mean
**SNA**	0.84	0.92	0.92	0.78	0.95	0.90
**SNB**	0.98	0.93	0.95	0.87	0.94	0.94
**ANB**	0.79	0.84	0.90	0.87	0.73	0.84
**FMIA**	0.82	0.78	0.70	0.78	0.84	0.79
**FMA**	0.90	0.93	0.75	0.94	0.95	0.91
**IMPA**	0.83	0.77	0.70	0.73	0.68	0.75
**I/SN**	0.73	0.80	0.73	0.69	0.65	0.72
**I/i**	0.79	0.82	0.82	0.69	0.55	0.75
**SN/Pocc**	0.92	0.79	0.91	0.83	0.71	0.85
**Fr/Pocc**	0.62	0.42	0.38	0.45	0.38	0.45
**Max/Mand**	0.96	0.92	0.93	0.96	0.86	0.93
**Mean**	0.87	0.84	0.83	0.82	0.81	-

^1^ LC-A: lateral cephalograms angles, obtained with straight line; ^2^ BPXR-: BPXR angles, obtained with straight line; ^3^ BPXR-PA: angles obtained from landmark acquisition, with the help of the PA; ^4^ LC-L: angles obtained from landmark acquisition on lateral cephalograms; ^5^ BPXR-L: angles obtained from landmark acquisition on BPXR lateral skull X-ray.

**Table 3:** Concordance with the LC-A method (reference method) estimated through the mean ICC of the three operators.

Angle	BPXR-A ^1^	BPXR-LPA ^2^	BPXR-L ^3^
**SNA**	0.72	0.80	0.75
**SNB**	0.87	0.88	0.83
**ANB**	0.83	0.76	0.71
**FMIA**	0.88	0.87	0.89
**FMA**	0.96	0.91	0.92
**IMPA**	0.80	0.76	0.70
**I/SN**	0.81	0.77	0.78
**I/i**	0.85	0.80	0.77
**SN/Pocc**	0.88	0.91	0.82
**Fr/Pocc**	0.72	0.66	0.73
**Max/Mand**	0.93	0.95	0.91
**Mean**	0.86	0.84	0.82

^1^ BPXR-: BPXR angles, obtained with straight line; ^2^ BPXR-PA: angles obtained from landmark acquisition, with the help of the PA; ^3^ BPXR-L: angles obtained from landmark acquisition on BPXR lateral skull X-ray.

## Data Availability

The data underlying this article will be shared at reasonable request to the corresponding author.

## Supplementary Materials

The following are available online at www.mdpi.com/article/10.3390/jcm10235477/s1, Figure S1: Intraoperator reliability for each landmark coordinate according to the method, Figure S2: Interoperator reliability of each landmark coordinate according to the method, Table S1: Results on distances.
