# Supplementary material for: Biplanar Low-Dose Radiograph Is Suitable for Cephalometric Analysis in Patients Requiring 3D Evaluation of the Whole Skeleton
Source: J Clin Med. 2021 Nov 23;10(23):5477. doi: 10.3390/jcm10235477 (PMC8658104; doi:10.3390/jcm10235477)
Supplement: Supplementary file 1 [file jcm-10-05477-s001.zip › Supplementary/Supplementary material 3.pdf]

Supplementary material 3

Table S1. Results on distances

	EOS-L		LC-L		Difference		
Distance	Mean	SD	Mean	SD	Mean	SD	p
S-N	68.62	3.93	68.93	3.35	0.22	1.41	0.60
N-ANS	53.29	3.79	54.55	3.04	1.24	2.33	0.20
ANS-PNS	55.14	6.32	56.57	5.17	1.75	2.0	0.22
Me-Ib	40.64	2.92	41.53	2.72	1.00	1.6	0.18
