# Supplementary figures and images for: Biplanar Low-Dose Radiograph Is Suitable for Cephalometric Analysis in Patients Requiring 3D Evaluation of the Whole Skeleton
Source: J Clin Med. 2021 Nov 23;10(23):5477. doi: 10.3390/jcm10235477 (PMC8658104; doi:10.3390/jcm10235477)

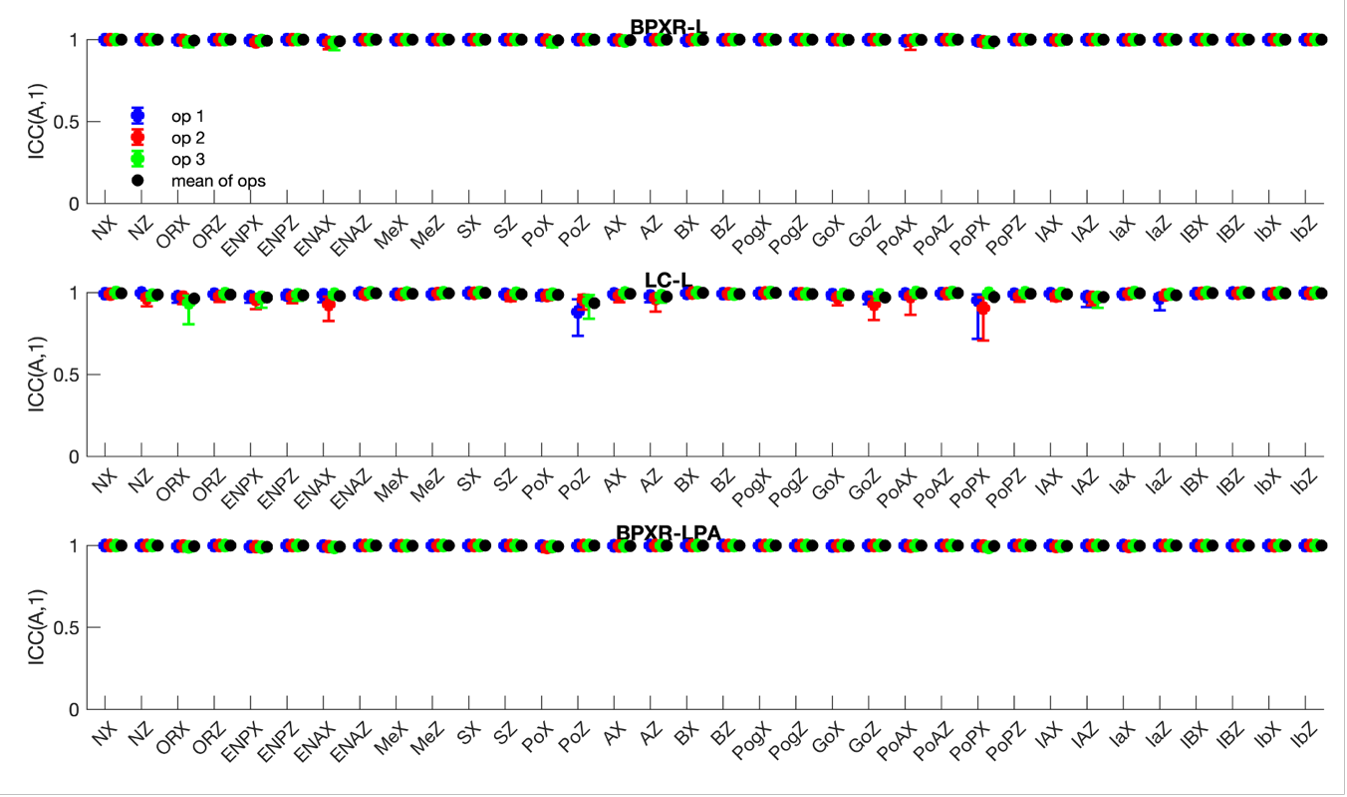

Supplement: Supplementary file 1 [file jcm-10-05477-s001.zip › Supplementary/Supplementary 1.tiff]

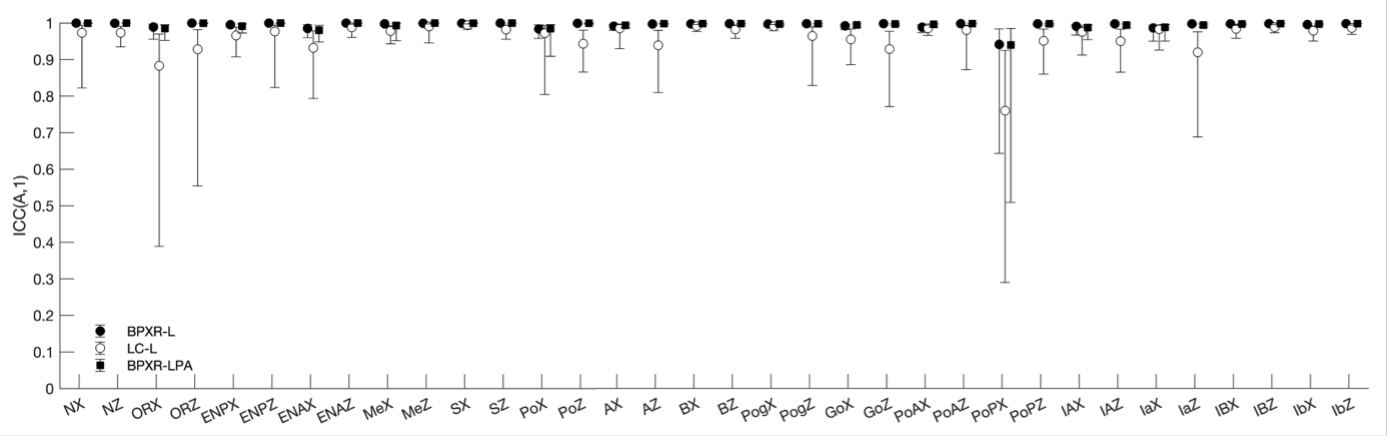

Supplement: Supplementary file 1 [file jcm-10-05477-s001.zip › Supplementary/Supplementary 2.tiff]
